# Supplementary material for: The Systems Biology Research Tool: evolvable open-source software
Source: BMC Syst Biol. 2008 Jun 29;2:55. doi: 10.1186/1752-0509-2-55 (PMC2446383; doi:10.1186/1752-0509-2-55)
Supplement: Additional file 1 — SBRT Archive. An archive of the current version of the Systems Biology Research Tool. [file 1752-0509-2-55-S1.zip › sbrt-1.4.0/doc/developers_guide/api/sbrt/kernel/fba/proc/CvFluxIntervalProcess.html]

CvFluxIntervalProcess


|  |  |  |
| --- | --- | --- |
| **Overview** \| **Package** \| **Class** \| **Use** \| **Tree** \| **Deprecated** \| **Index** \| **Help** | |  |
| **PREV CLASS**   **NEXT CLASS** | **FRAMES**    **NO FRAMES**     **All Classes** |  |
| SUMMARY: NESTED \| FIELD \| CONSTR \| METHOD | DETAIL: FIELD \| CONSTR \| METHOD |  |


---


## sbrt.kernel.fba.proc Class CvFluxIntervalProcess

```
java.lang.Object
  sbrt.kernel.fba.proc.FbaProcess<R>
      sbrt.kernel.fba.proc.CvFbaOptProcess<java.util.Map<java.lang.String,Interval>>
          sbrt.kernel.fba.proc.CvFluxIntervalProcess
```

**All Implemented Interfaces:**: KernelProcess<java.util.Map<java.lang.String,Interval>>, MonitoredProcess<java.util.Map<java.lang.String,Interval>>

---

``` public final class CvFluxIntervalProcess extends CvFbaOptProcess<java.util.Map<java.lang.String,Interval>> implements MonitoredProcess<java.util.Map<java.lang.String,Interval>> ```

This class is used to perform constraint variation flux interval experiments.

**Author:**
:   This class was written and documented by Jeremiah Wright while in the
    Wagner lab.

---

**Constructor Summary**

`CvFluxIntervalProcess(FluxIntervalComputer computer)`
  
          Constructs a new constraint variation flux interval experiment.


| **Method Summary** | |
| --- | --- |
| `Fluxome` | `getFluxome()`             Returns the fluxome used in this experiment. |
| `ProgressMonitor` | `getProgress()`             Returns a progress monitor for this experiment. |
| `void` | `run(ResultsManager<java.util.Map<java.lang.String,Interval>> resultsManager)`             Runs this process and adds the generated results to the provided results manager via its `ResultsManager.addData(Object)` method. |
| `void` | `setConstraintSource(java.lang.Iterable<FluxConstraints> constraintSource)`             Sets the constraint variation input source. |
| `void` | `setReactionNames(java.util.Collection<java.lang.String> reactionNames)`             Sets the names of reactions for which flux intervals are to be computed. |

**Methods inherited from class sbrt.kernel.fba.proc.CvFbaOptProcess**

`restoreConstraints, setConstraints`

**Methods inherited from class sbrt.kernel.fba.proc.FbaProcess**

`toString`

**Methods inherited from class java.lang.Object**

`clone, equals, finalize, getClass, hashCode, notify, notifyAll, wait, wait, wait`

**Constructor Detail**

### CvFluxIntervalProcess

```
public CvFluxIntervalProcess(FluxIntervalComputer computer)
```

:   Constructs a new constraint variation flux interval experiment.

    **Parameters:**: `computer` - the computer with which all flux intervals will be computed.


**Method Detail**

### getFluxome

```
public Fluxome getFluxome()
```

:   Returns the fluxome used in this experiment.

    :   **Specified by:**: `getFluxome` in class `FbaProcess<java.util.Map<java.lang.String,Interval>>`
    :   **Returns:**: the fluxome used in this experiment.

---


### getProgress

```
public ProgressMonitor getProgress()
```

:   Returns a progress monitor for this experiment.

    :   **Specified by:**: `getProgress` in interface `MonitoredProcess<java.util.Map<java.lang.String,Interval>>`
    :   **Returns:**: a progress monitor for this experiment.

---


### setConstraintSource

```
public void setConstraintSource(java.lang.Iterable<FluxConstraints> constraintSource)
```

:   Sets the constraint variation input source.

    :   **Parameters:**: `constraintSource` - a source of constraint sets whose effect upon application is to be computed.

---


### setReactionNames

```
public void setReactionNames(java.util.Collection<java.lang.String> reactionNames)
```

:   Sets the names of reactions for which flux intervals are to be computed.

    :   **Parameters:**: `reactionNames` - the names of reactions for which flux intervals are to be computed.

---


### run

```
public void run(ResultsManager<java.util.Map<java.lang.String,Interval>> resultsManager)
```

:   Runs this process and adds the generated results to the provided results
    manager via its `ResultsManager.addData(Object)` method.

    First, a set of flux constraints is retrieved from the constraint source
    and applied. Then the flux intervals of the reactions with the current names
    are computed and added to the provided results manager. Finally, the
    flux constraints are restored to their original values. This process
    is repeated until the end of the constraint source is reached.

    :   **Specified by:**: `run` in interface `KernelProcess<java.util.Map<java.lang.String,Interval>>`
    :   **Parameters:**: `resultsManager` - the results manager to which the results will be added.


---


|  |  |  |
| --- | --- | --- |
| **Overview** \| **Package** \| **Class** \| **Use** \| **Tree** \| **Deprecated** \| **Index** \| **Help** | |  |
| **PREV CLASS**   **NEXT CLASS** | **FRAMES**    **NO FRAMES**     **All Classes** |  |
| SUMMARY: NESTED \| FIELD \| CONSTR \| METHOD | DETAIL: FIELD \| CONSTR \| METHOD |  |


---
